# A holistic adaptive ageing framework (HAAF) to address complex challenges in ageing

**DOI:** 10.1007/s00391-025-02467-9

**Published:** 2025-07-14

**Authors:** Kaarin J. Anstey

**Affiliations:** School of Psychology, University of New South Wales, NSW 2052 Sydney, Australia

**Keywords:** Adaptive ageing, Framework, Technology, Social policy, Multidisciplinary approaches, Adaptives Altern, Rahmen, Technologie, Sozialpolitik, Multidisziplinäre Ansätze

## Abstract

Many of the challenges facing ageing societies involve an interaction of factors from many domains and levels, including person-level age-related changes in function through to the physical environment, economy, urban design and social policies. The pace of change in our societies is accelerated by climate change and the rapid introduction of artificial intelligence and other technologies. When presented with practical challenges or problems to solve that are inherently due to an ageing society, it is not possible for any individual to have the expertise and capacity to address all the dimensions involved. Therefore, developing a multidisciplinary framework that signals the dimensions of influence that need to be considered in adaptive ageing, may assist in optimizing how complex issues in ageing are tackled. This paper proposes a holistic adaptive ageing framework (HAAF) as a starting point for multidisciplinary and multisectoral approaches to develop optimal solutions to complex problems. The five dimensions of the framework include lived experience, person-level adaptation, environmental context, technology and social and economic policy. Future research is needed to operationalize the framework and evaluate its application to complex problems.

## Abstract (de)

Viele der Herausforderungen, mit denen sich alternde Gesellschaften konfrontiert sehen, beinhalten ein Zusammenspiel von Faktoren aus vielen Bereichen und auf zahlreichen Ebenen, von altersbedingten Funktionsänderungen des alternden Menschen bis hin zu Umwelt, Wirtschaft, Stadtplanung und Sozialpolitik. Das Tempo des Wandels in unseren Gesellschaften wird durch den Klimawandel und die rasche Einführung von künstlicher Intelligenz und anderen Technologien beschleunigt. Bei der Bewältigung praktischer Herausforderungen oder Probleme, die sich aus der Alterung der Gesellschaft ergeben, kann ein Einzelner nicht über das Fachwissen und die Fähigkeit verfügen, alle damit verbundenen Aspekte zu berücksichtigen. Die Entwicklung eines multidisziplinären Frameworks, das die Einflussdimensionen aufzeigt, die beim adaptiven Altern berücksichtigt werden müssen, kann dazu beitragen, die Bewältigung dieser komplexer Fragen zu optimieren. In diesem Beitrag wird ein ganzheitliches Rahmenprogramm für adaptives Altern als Ausgangspunkt für multidisziplinäre und sektorübergreifende Ansätze zur Entwicklung optimaler Lösungen vorgeschlagen. Zu den fünf Dimensionen gehören die Lebenserfahrung, die Anpassung auf der Ebene des Einzelnen, der Umweltkontext, die Technologie sowie die Sozial- und Wirtschaftspolitik. Zukünftige Forschung ist erforderlich, um den Rahmen zu operationalisieren und seine Anwendung auf komplexe Probleme zu bewerten.

## Introduction

Many of the issues that arise as individuals age require complex solutions that need to consider both their personal circumstances and capacities, and the social systems in which they live; however, many academic gerontologists and scientists work within individual disciplines or a group of similar disciplines. It is argued here that there is a need for gerontology to establish a more comprehensive, multidisciplinary framework for thinking about ageing that will enable academic and scientific experts, and private and public sectors to work together to solve real-world problems. This will assist in applying academic and scientific expertise to solving the real-world challenges facing us in creating optimal societies for people of all ages.

Adaptive ageing is a concept that can be applied to both individuals and societies and provides a useful starting point for framing a holistic, interdisciplinary approach to solving challenges in the real world. A holistic approach to adaptive ageing can incorporate both the dynamics of ageing at multiple levels within the individual as well as the adaptive processes at the societal level and also bring these two domains of the individual and society together. Technology is also integral in enabling both personal adaptation to age-related changes as well as society’s adaptation to an increasingly older population. This article therefore proposes the first steps towards a broad interdisciplinary framework for adaptive ageing.

## Why we need a new framework of holistic adaptive ageing

Problems and challenges in real life do not fall into academic discipline categories and often have economic, cultural and situational determinants and constraints. Without an intentional plan to address adaptive ageing, there is an increased likelihood that new technologies, for example, are not designed to accommodate the sensory and cognitive changes that occur in later life or that opportunities to increase social connection will be missed due to suboptimal urban design. Therefore, there is a need for an expanded framework of adaptive ageing that is fit for purpose in addressing complex issues of ageing. This will need to consider the core areas of ageing science which are often not recognized in broader fields of technology design and urban design. For example, with an ageing population, we have increasing numbers of people with individual sensory and cognitive issues to which the individual needs to adapt [1]. In normal ageing there is a decline in the useful field of view, visual acuity and contrast sensitivity [30, 34] and an increase in the prevalence of age-related eye diseases, such as cataract and macular degeneration [18]. With brain ageing there are well described cognitive changes in memory and executive function, slowing of reaction time etc. [21, 23, 28].

At the societal level, an adaptive and responsive policy context which is informed by knowledge of biological ageing and which utilizes new developments in artificial intelligence and sensor technology is needed. This in turn needs to consider familial and social structures such as families, communities and paid and unpaid caregivers. Without seeing all the components involved in a problem or challenge, there is a risk that one part will be left out and a suboptimal solution to care needs will be provided. For example, the lived experience of the person with dementia is vital for providing feedback on whether needs are being met but if omitted then a system may be designed that they cannot use or does not meet their needs. If an economic evaluation is not undertaken, solutions may not be cost-effective or feasible. A system may be designed that is functional in the test laboratory but which ignores cultural sensitivities related to gender or ethnicity and this may reduce its uptake and effectiveness. In many contexts, the problems we face are also occurring in the context of regulation, both legal and professional. Although we are generally aware of this complexity as we encounter it in our daily lives, it may be beneficial to create a more formalized framework that is consciously and pre-emptively applied to solving problems related to ageing. Here I argue that if we apply an adaptive ageing framework which systematically identifies the different elements of a situation or problem to be addressed (or which do not need to be addressed), then we can manage the complexity of situations we face in a more efficient and effective manner that respects the different people and elements involved. The advantage of a wide, multilevel, interdisciplinary framework to think about adaptive ageing, is that it enables us to incorporate different levels and domains of influences on the individual as well as on society.

## Advantages of a broader interdisciplinary adaptive ageing framework

Consciously employing a framework of adaptive ageing will enable teams with a range of expertise to conceptualize and work across the complex and multilevel influences on ageing to address complex challenges such as designing housing and healthcare policies for care provision and transport. A challenge facing all communities is how to enable people to age at home at increasingly older ages, in the context of climate change. Addressing this involves understanding the physiological impact of climate events and conditions on older adults as well as home design, social networks and services. With a declining dependency ratio, technological innovation is required to provide support and services for older adults.

A common framework for communicating about adaptive ageing, will facilitate the development of discourse between quite disparate areas of technical and intellectual expertise so that people working in these areas can come together and focus on a common goal. Focussing on the individuals making adaptations to their own sensory and cognitive changes is important for improving their quality of life at an individual level but is not sufficient for providing for their needs within a complex network of care services in a specific physical and cultural context.

## Prior uses of the term adaptive ageing within theories of late-life development

Many uses of the concept of adaptive ageing within gerontological theories are discipline specific, focusing either on the individual or biological processes [6, 8, 10, 13, 29]. Adaptive ageing is a concept that has been referred to in descriptions of successful ageing [31] and in neurobiological theories to explain neuroplasticity [8]. Adaptation has been incorporated into major theories of human development such as the selective optimization and compensation theory by Baltes which considers how psychological mechanisms of adaptation can compensate for deficits associated with cognitive and sensory declines [5]. Adaptation is also integral to theories of social and emotional development in late life [10] and the continuity theory [2].

Within the fields of biology and neuroscience, adaptation in ageing is generally viewed as innate changes in response to biological ageing that enable the organism to maintain function or homeostasis. For example, metabolic changes may occur in the ageing brain, mediated by insulin growth factor, sirtuin enzymes and mTOR, which is a protein that controls cellular metabolism and other functions. In the ageing brain, neural adaptation occurs to compensate for age-related changes and this may be seen in functional magnetic resonance imaging studies (fMRI) as greater recruitment of brain networks [14] and reduction in neural excitement [1].

The concept of adaptive ageing is invoked in the field of socioemotional ageing to explain the ageing paradox, whereby older adults appear to have an increase in well-being, despite a decline in physical and cognitive capacities [11]. Here, within the socioemotional selectivity theory [9], adaptation describes the process by which older adults become more selective with their emotional goals and relationships to minimize stress and increase well-being. Another related theory in which adaptation is characterized in the sphere of socioemotional ageing is that of self-determination. In this theory, adaptations describe the changes that older adults make to deal with age-related challenges. These adaptations may include things like self-acceptance (of age-related changes), maintaining a healthy lifestyle, using alternative transport, learning and participation in social activities. Altogether, these facilitate self-determination which is characterized by autonomy, a sense of competence and relatedness [7].

Societal adaptation to ageing has also been broadly evaluated in terms of policy changes that address dimensions of societal preparedness for supporting an increasingly older population. The Aging Society Index rates countries in terms of productivity and engagement, well-being, equity, cohesion and Security [12]. This offers an important method of comparing countries and measuring adaptation within specific domains but does not connect individual level changes to societal changes. Using the concept of adaptation as a conscious strategy by which societies seek to optimize the benefits of increased longevity is implied by authors who review societal adaptation [12].

## A holistic adaptive ageing framework

**Fig. 1:**
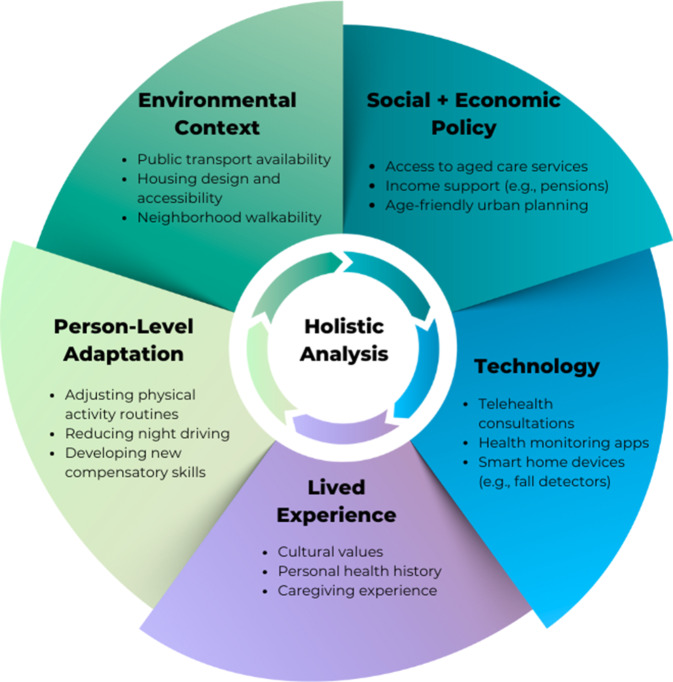
Schematic of dimensions for a holistic adaptive ageing framework. Note. Bullet points are examples and not exhaustive lists

The ideal adaptive ageing framework is one that allows us to conceptualize how different contextual and societal changes and influences will impact ageing individuals and ageing societies as well as how they are impacted by them. This differs from a systems approach that focuses on the complex systems involved in individual ageing [19] and the societal level metrics [12] because it addresses both the societal level and individual level challenges related to ageing. However, some of the key points relating to complex systems also apply to an adaptive ageing framework, for example, the observation that complex systems have emergent properties. The contemporary holistic framework for adaptive ageing (HAAF) is presented here to provoke thinking about adaptive ageing as a way for individuals, academics, policy makers and technology producers to reap the benefits of increased longevity and proactively plan for it. The HAAF has five dimensions which collectively capture the domains and perspectives needed to design solutions that fit with contextual constraints and meet the needs of individuals and society (Fig. 1).

### a) Lived experience.

The subjective experience of the individual is implied in many psychological models of adaptation and is increasingly being measured and incorporated into research designs. When developing a solution to a real-world problem, obtaining insights from people involved and their experiences is now considered an essential step. Research in mental health for example, has found that involvement of people with lived experience led to more relevant outcome measures being included in studies [24]. It is essential that programs and interventions are acceptable, accessible and designed for the people they are targeting. This may apply to designing solutions to ageing in place, aged care, mobility and optimal financial products; however, using person-centered design and lived experience alone is insufficient for developing sophisticated and evidence-based solutions and does not provide the scientific knowledge needed to develop solutions.

### b) Person-level adaptation to age-related changes

(incorporating both losses and gains) through behavioral and cognitive changes is the second dimension of adaptive ageing. Intricate knowledge of the physiological, cognitive and sensory changes that occur in normal ageing and with age-related disease is a key area of expertise that needs to be drawn on when designing solutions for older adults. This dimension focuses on the individuals’ capacity to adapt to intrinsic age-related changes and declines as well as external changes that interact with physiological ageing. These changes include cognitive, affective and physical changes within an individual and include beliefs and attitudes. A key basis for this dimension is the theory of adaptation formulated in terms of selective optimization and compensation, proposed by Baltes [4] that incorporate gains and losses. Examples of person-level changes include adaptation, including making changes to one’s physical activity patterns, to align with age-related changes in endurance, flexibility and muscle strength or reducing night driving as normal vision with ageing declines at night. Adaptive ageing may involve developing new compensatory skills, drawing on external resources and aids or compensating through substitution of resources or abilities [33]. It may also involve or be influenced by attitudes and control beliefs [15, 16] which limit or promote individual adaptation [32]. Person-level adaptation to age-related changes is an area that is the focus of many healthcare professionals such as psychologists, occupational therapists and physiotherapists and requires knowledge of the underlying physiological and cognitive changes in ageing as well as neuroplasticity and the capacity to change or modify activities.

### c) Using technology to optimize adaptation to age-related changes.

A third dimension of adaptive ageing is the use of technology (e.g., to compensate for sensory loss, reduction in mobility) [25]. Traditional examples may be sensory aids such as corrective glasses and hearing aids, and more recent examples include e‑health services, artificial intelligence (AI) including generative AI and mobile technology. With transition to Industry 4.0 well underway [20] there is a need to incorporate technology into our frameworks and to also consider how the design of technology can be adapted to meet the needs to ageing adults. With population ageing leading to increases in care and health costs at the same time as the number of people of working age declines, technology is a key mechanism for implementing adaptive strategies to enable older adults meet their own needs and for care and services to be delivered.

### d) Social and economic policies and structures

are the fourth dimension of the HAAF and refer to context-level adaptation in a holistic approach to adaptive ageing, we need to enable ageing well through creating socioeconomic conditions or opportunities for social participation for ageing adults. We also need to consider the constraints and opportunities created by economic circumstances, as well as social policy as key influences on the types of solutions possible for addressing complex problems. Having economic and social policies supporting an age friendly society will facilitate engagement by more ageing adults regardless of mobility limitations. Examples of social and economic policies are those relating to income support and care provision, urban design and transport systems. In countries where older adults fund their own retirement through superannuation and savings, such as Australia (alongside a government pension), there is an increasing need for policies to support sound financial decision-making at the individual level, the development of products that meet the needs of diverse consumers, and responsible practices at the corporate level [17, 22]. Behavioral economics theories seek to explain how human behavior and financial decision making can be predicted and adapted to optimize outcomes for ageing individuals and society.

### e) The physical environment

is the fifth dimension that needs to be considered within the HAAF. Practical aspects of local geography and climate influences health and well-being and may interact with person level changes in ageing. For example, older adults are more susceptible to heat-related morbidity and mortality [3, 26, 27]. With climate change and increasing urbanization, changes to the environment are inevitable. The physical environment including temperature, pollution, urban density, access to green and blue spaces, and an individual’s local physical environment (their home, room, suburb), all play a role within a model of adaptive ageing.

Drawing together these five dimensions systematically, in any evaluation of a situation or challenge, will enable a holistic ecological analysis and articulation of a project or problem in the first instance. Researchers work within specific disciplines and may for example be interested in assessing their domain of interest in relation to one other domain (e.g. examining cognition and how cognition impacts driving); however, for tackling the larger challenges of an ageing society, we need a more thorough analysis of the dimensions of adaptive ageing, and to bring our expert knowledge of biological ageing, lived experience, systems of care, the built environment and economics. Having a well-developed framework will help in the application of academic expertise and signal which experts are needed to contribute to overall solutions.

## Application and implementation of the holistic adaptive ageing framework

The HAAF is relevant to gerontological research from at least two perspectives. The first is to contextualise discipline-specific projects within a wider context so that they are more accessible to a wider audience. A project may be conducted to investigate a specific topic, such as the effect of hearing loss on social isolation but the findings may be interpreted at the level of the individual as well as considering other dimensions of the HAAF, such as the role of technology in addressing the problem, social factors such as stigma, policy influences such as funding models for hearing supports. This recognizes that when focusing on specific topics, they occur in a complex and dynamic environment that has multiple influences. Research that focusses on understanding why older adults with hearing loss have low adoption of hearing aids may use a variety of person-centered methodologies such as focus groups, in-depth interviews and surveys to uncover and understand the reasons for this low uptake. Viewed within the wider framework, the findings of the study could be interpreted in the context of cultural, economic and logistical factors that influence access and uptake.

The second perspective where the HAAF is relevant is when gerontologists work as part of a multidisciplinary team to address large scale societal challenges. For example, designing housing or villages for older adults that will be accessible and adaptable as they age and conducting research on identifying the features of the most cost-effective design with the best health and psychosocial outcomes. Optimal design requires considerations of all dimensions of the HAAF, and applying the framework at the outset may prompt consideration of all the key variables that will influence outcomes to ensure that optimal ageing is supported.

**Fig. 2:**
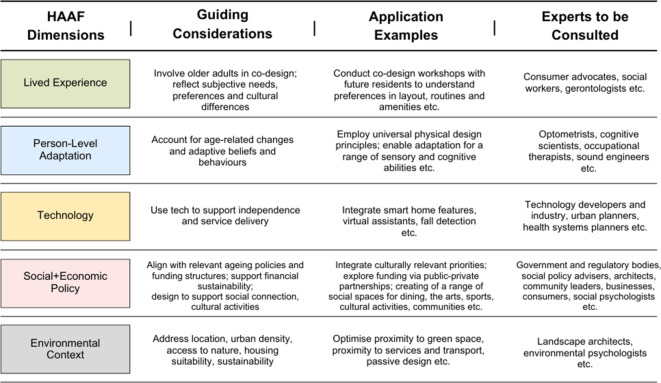
Example of indicative application of HAAF matrix in planning new urban development

In practice, application of the framework to a discipline-specific project may occur at the design stage or the discussion of research findings. The application of the HAAF framework to larger research challenges will be more powerful. Having a formal matrix that identifies key stakeholders and potential influences on the project outcomes at the project planning stage will enable assembly of the coalition of experts and methodologies required to deliver an optimal solution. For example, the views and preferences of potential end users and residents of a housing development would be considered, alongside cultural, economic, environmental and regulatory factors. Detailed characterization of the components of a design solution, with measurements taken longitudinally, will enable evaluation of how factors impact outcomes for healthy ageing. The characterization of elements of the framework for designing residential environments and the activities that are involved are shown in an indicative matrix (Fig. 2) and could include:

### Dimension a—Lived experience:

interviews with potential residents to understand their daily patterns and preferences, needs for social engagement and mobility, life space, cultural and religious practices, employment and recreation. Collaborations could therefore occur between end users and the project team.

### Dimension b—Personal level adaptation to age-related changes:

evaluation of the impact of sensory, cognitive and physical limitations and impairments on the use of space, needs and design of residences which may impact things like lighting, access, signage and complexity of structures. Collaborations may occur between academic experts in aspects of human ageing such as psychologists, vision scientists, audiologists, geriatricians and exercise physiologists as well as designers, technology companies and engineers.

### Dimension c—Using technology to optimize adaptation to age-related changes:

identification and evaluation of optimal assistive technology, sensors, and robots for individual settings as well as to facilitate communication, transport, and daily activity which is also environmentally sustainable. Technology used by people to improve their lifestyle (e.g. wearables that monitor heart health, physical activity, noise exposure etc.), communication (e.g. digital platforms in the home to engage with health providers and family, AI companions, virtual delivery of health and care services, social networking). Collaborations may occur between technology developers and industry, urban planners, healthcare systems planners and communications companies.

### Dimension d—Social and economic policies and structures:

documentation of relevant policy and regulations that impact funding and activities. Collaborations may occur between different levels of government and regulatory bodies, social policy advisers, to ensure implementation of the project.

### Dimension e—Physical environment:

surveying the physical environment to ensure that the residential design is adaptive and appropriate to climate, exposure to pollution, potential climate risk, and other local environmental characteristics. Emphasizing exposure to beneficial geographical characteristics such as green and blue spaces. Ensuring environmental factors are considered in the design will require collaboration with experts in environment and health.

Researchers working within the HAAF could embed measures that can be repeated over time to enable analysis of the effectiveness of designs that aim to optimize ageing well. The measures could be related to meaningful outcomes for individuals and society. For individuals this could include cognitive health, mental wellbeing, longevity and quality of social engagement. Meaningful outcomes for society could be changes in health service use and associated costs (or cost savings), the number of ageing adults retained in the workforce (with accompanying economic benefits), increased social cohesion and intergenerational activities.

## Practical conclusion

Systematic multidisciplinary approaches that consider the five dimensions described above, are needed to address the common complex challenges of ageing. Using this framework will allow experts to work together in applying very specific technical expertise, such as knowledge of age-related sensory loss or urban design. The types of situations and problems we urgently need to tackle collectively include optimizing how people can remain in productive employment longer, how they can age at home or the place of their choosing, how to design cost-effective care services for people living with dementia while we have a diminishing working age population and how to design financial advice and systems that support ageing adults once they leave paid work. Future research is needed to operationalize and refine the framework and apply it in the real world.

## Declarations

This article does not contain any studies on human or animal subjects.
